# Long-Wavelength Infrared Sensing by Cytochrome C Protein Thin Film Deposited by the Spin Coating Method

**DOI:** 10.3390/s131115833

**Published:** 2013-11-20

**Authors:** Bo-Yu Lai, Chung-Hao Chu, Guo-Dung John Su

**Affiliations:** Graduate Institute of Photonics and Optoelectronics, National Taiwan University, No. 1, Roosevelt Road, Section 4, Taipei 10617, Taiwan; E-Mails: niecday@gmail.com (B.-Y.L.); r98941023@ntu.edu.tw (C.-H.C.)

**Keywords:** bolometer, cytochrome c protein, long-wavelength infrared, temperature coefficient of resistance

## Abstract

High infrared absorption, large temperature coefficient of resistance (TCR) and small 1/f noise are preferred characteristics for sensing materials used in bolometers. In this paper, we discuss a cytochrome c protein as a potential sensing material for long-wavelength bolometers. We simulated and experimentally proved high infrared absorption of cytochrome c in the wavelength between 8 μm and 14 μm. Cytochrome c thin films were deposited on a hydrophilic surface using the spin coating method. The resistance variation with temperature is measured and we show that the TCR of cytochrome c thin films is consistently higher than 20%. The measured values of 1/f noise were as low as 2.33 × 10^−13^ V^2^/Hz at 60 Hz. Finally, we test the reliability of cytochrome c by measuring the resistance changes over time under varying conditions. We found that cytochrome c thin films deteriorated significantly without appropriate packaging.

## 1. Introduction

It is possible to operate thermal detectors at room temperature without cryogenic devices. Thermal detectors are advantageous due to their low cost and weight, while the response is relatively slow and responsivity is lower than that of photon detectors. Therefore, they have potential for portable applications. There are several thermal detectors: pyroelectric [1], thermopile [2], and bolometers [3,4]. The operating principle of a bolometer is that when sensing materials absorb infrared radiation and turn the radiation into heat, the resistance of the sensing materials will change due to the rising temperature. This resistance change is then measured and processed into temperatures which can be used to create an image. To obtain a better response, the resistance difference of sensing materials should be as large as possible. Therefore, it is important to find sensing materials with a high temperature coefficient of resistance (TCR).

Popular sensing materials in use for bolometers include vanadium oxide (VO_x_) and amorphous silicon (a-Si). The TCR values of vanadium oxide and amorphous silicon are reported to be −4%/K [5] and −3%/K [6], respectively. In addition, other materials are being tudied. Titanium is a metal material that is used in microbolometers and has a TCR of approximately 0.35%/K [7]. The superconducting material yttrium barium copper oxide (YBaCuO) is reported to have a TCR of approximately −4%/K [8]. However, semiconductor materials require costly vacuum deposition tools, such as chemical vapor deposition or pulse laser deposition.

Researchers are also investigating organic materials. Polymeric conducting materials and biomaterials have been investigated and studied. A poly(3,4-ethylenedioxythiophene):poly(styrenesulfonate) (PEDOT:PSS) thin film was reported to have a TCR of over −4%/K [9]. Yavuz and Aldissi examined the potential of using proteins as a microbolometer sensing material [10]. Deb reported that cytochrome c protein on the top of silicon-dioxide could obtain a TCR of over 20%/K [11,12]. These materials present high TCR measurements and can be deposited by a self-assembly monolayer (SAM) method, Langmuir-Blodgett (LB) method and adsorption, which is quicker and cheaper than the traditional semiconductor deposition techniques.

In this paper, we study the cytochrome c protein further due to its high TCR and commercial availability. In addition, we demonstrate that it can be deposited in a room environment without the requirement for an expensive vacuum system. We first show the high infrared absorption rate of the cytochrome c protein in the long wavelength region between 8 μm and 14 μm. We then develop a spin coating process to deposit protein thin film on a hydrophilic surface of SU-8 photoresist. It gave a high TCR and low 1/f noise. The reliability of the cytochrome c protein in ambient environments and sealed packages is then tested. It is shown that the cytochrome c protein has great promise for making a novel bolometer if the appropriate packaging is considered.

## 2. Long-Wavelength Infrared Absorption of the Cytochrome C Protein

When infrared radiation passes through a sample, certain frequencies are absorbed by the molecules of the substance, causing molecular vibrations. Complex molecules have lots of bonds, and vibrations can be conjugated, leading to infrared absorption at characteristic frequencies that may be associated to the chemical groups. We are interested in the long wavelength infrared (LWIR) range (8∼12 μm) since this band is valuable for detecting a room temperature object.

**Figure 1.**
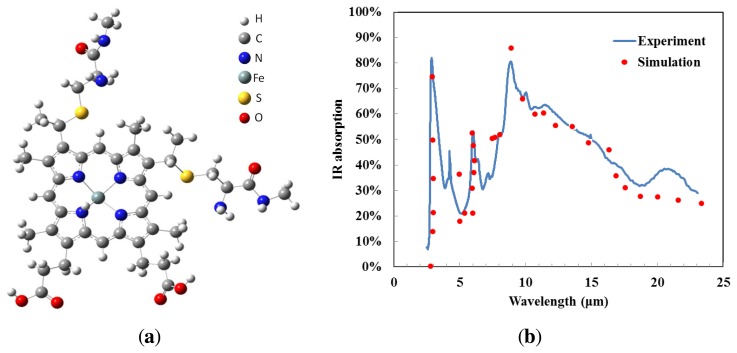
(**a**) The molecular structure of cytochrome c in GaussView^®^. (**b**) The infrared absorption of the cytochrome c protein by software simulations and experimental measurements.

Gaussian^®^ and GaussView^®^ are examples of electronic structure modeling software, and were used to simulate the expected infrared absorption spectrum of cytochrome c. It is assumed that electromagnetic radiation is absorbed due to molecular vibration; therefore, the molecular structure of cytochrome c must be investigated. We used the molecule building module in GaussView^®^ to create the 3D molecular structure, as shown in Figure 1a. Gaussian^®^ approximates orbital shapes and orbital energies of a given molecular geometry using model chemistry. We selected the Hartree-Fock method to perform our frequency analysis. The vibrational frequencies and infrared spectra of the molecule were displayed by GaussView^®^ after the Gaussian^®^ run completed, resulting in the vibrational frequency calculation shown as dots in Figure 1b. When molecular vibrations corresponded to a certain frequency, infrared radiation was absorbed. Due to the complicated nature of the molecular structure of cytochrome c the infrared absorption simulation results showed multiple absorption wavelengths. The IR absorption of cytochrome c is relatively high in the regions 3 μm and 5–11 μm. There are numerous molecular vibrational modes. Cytochrome c shows good absorption due to C–C bonds, C–O bonds and C–N bonds [13], and no additional material is required to enhance absorption for infrared spectral bands. This makes cytochrome c protein a promising material for uncooled thermal detectors.

We measured the infrared absorption of cytochrome c thin films from the wavelength of 2.5 μm to 23 μm using Fourier transform infrared spectroscopy (Thermo Nicolet Nexus 470 FTIR). The sample was a mixture of 2 mg cytochrome c and 5 mg potassium bromide (FTIR grade KBr, from Sigma Aldrich, St. Louis, MO, USA) [14]. The mixture was ground to a fine powder using a mortar and pestle, and a disc (0.1 mm thick and 10 mm in diameter) was formed after a pellet press.

The sample was placed in the 470 FTIR spectrometer. The FTIR spectrometer has two types of detectors: deuterated triglycine sulfate (DTGS) and mercuric cadmium telluride (MCT/A). We used the MCT/A detector because it provides superior sensitivity and speed, however, this detector requires liquid nitrogen. The IR spectra were measured by passing infrared light through the protein discs and recording what fractions of incident radiation are absorbed at particular energies. The energy of a peak in the spectrum corresponds to the vibration frequency of part of the sample.

The incident IR radiation was absorbed by cytochrome c. The IR absorption spectrum of the cytochrome c protein thin film, in the wavelength range between 2.5 μm and 23 μm, is given as the solid line in Figure 1b. The spectrum indicates that there are two typical absorption peaks in the working band of mid- and long-wavelength IR detectors. The IR absorption is seen to exceed 50% in two regions: 3–4 μm and 8–14 μm. We conclude that the cytochrome c film has a high absorption coefficient in mid- and long-wavelength IR, which enhances the detector performance. The experimental data matches the simulation results closely. The experimental results confirm that the high infrared absorption of the cytochrome c protein could be due to the molecular structure and the diversity of vibration frequencies.

## 3. Spin Coating Cytochrome on a Hydrophilic Surface

The cytochrome c protein was stored in crystalline powder form. The authors purchased it from Sigma Aldrich (C2506, ≥95% (SDS-PAGE), from equine heart) and used it directly without any further purification. The protein solution was prepared by mixing the cytochrome c protein with a phosphate buffer solution and de-ionized (DI) water. We prepared the phosphate solution by adding K_2_HPO_3_ and KH_2_PO_3_ in the water. The phosphate solution had a concentration of 0.1 M at pH 6.8. They were mixed with a ratio of 2 mg cytochrome c, 1 mL phosphate buffer solution, and 1 mL DI water.

**Figure 2.**
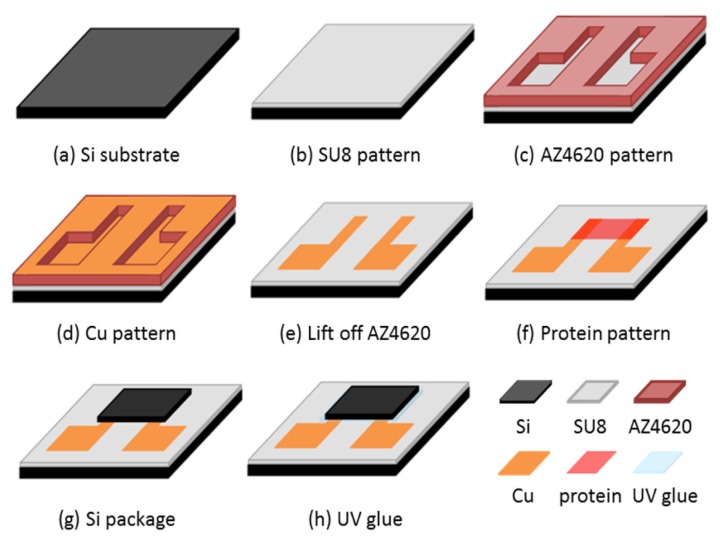
Schematic diagram of the test chip fabrication.

The test chip preparation is shown in Figure 2. We used a 2 cm × 2 cm silicon chip as a substrate. The diluted negative photoresist SU-8 was used as an electrical insulation layer for protein deposition. The diluted SU-8 photoresist was made by adding SU-8 thinner to SU-8 photoresist at a weight ratio of 6:10. The diluted SU-8 photoresist was spun at 500 rpm for 5 s and then 2,500 rpm for 30 s. The thickness was approximately 8 μm. The exposure time was 10 s with a 10 mw/cm^2^ UV light source.

The SU-8 thin film was hard-baked at 100 °C for 3 min after developing. We used a lift-off process to deposit the copper pattern. The photoresist mask was AZ4620. The AZ4620 was spun at 2,500 rpm to achieve sufficient thickness for the lift-off process. We then deposited the copper layer by thermal evaporation and lifted off the AZ4620 layer by immersing in acetone. The patterned electrodes were separated. The thickness of the copper electrodes was approximately 1,000 nm. The chip was then subjected to a UV/ozone process to change its hydrophobicity. The protein solution was dropped on the chip, which remained static for 60 s, and was then spin-coated at 600 rpm for 10 s. Finally, we dried the chip for 4 h in a vacuum glove box (∼10^−2^ Torr). The chip was then packaged using silicon and UV glue with 5 min exposure under a UV lamp.

**Figure 3.**
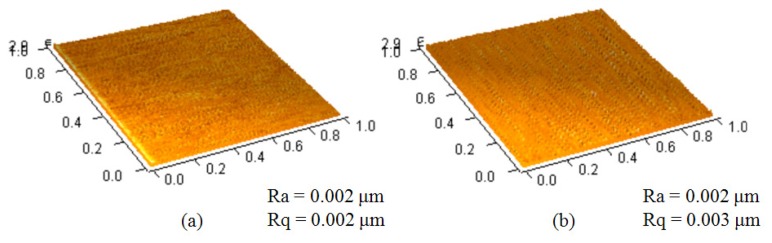
Atomic force microscope (AFM, OBJ-204C, ITRI of Taiwan) images of the SU-8 photoresist (**a**) before and (**b**) after UV/ozone treatment. All images were taken at a scan size of 1 × 1 μm^2^ with a 128 × 128 pixel^2^.

We chose SU-8 photoresist as a substrate layer to deposit the cytochrome c protein since the hydrophobicity of SU-8 photoresist could be changed by treating the epoxy functional group [15]. There are reports showing how to change the epoxy functional group into others, such as an amino [16] or thiol functional group [17]. The UV/ozone process turns the epoxy functional group of the SU-8 photoresist surface into C=O and the phenol group (benzene-OH) [18], which are highly polar overall. Since cytochrome c has a calculated dipole moment greater than 300 Debye [19], we assumed it can be attached to an SU-8 photoresist by dipole-dipole interaction. We changed the UV/ozone process time to control the hydrophobicity of the SU-8 photoresist. The lamp of the UV/ozone cleaner (model UV-1, Samco) is 110 W at a wavelength between 254 nm and 182 nm. The oxygen flow rate is 0.5 L/min. We used an atomic force microscope (AFM, OBJ-204C, ITRI, Hsinchu, Taiwan) to measure the surface roughness of the SU-8 photoresist before and after UV/ozone treatment for four minutes. The images were all taken at a scan size of 1 × 1 μm^2^ with a 128 × 128 pixel^2^. As can be seen from the measurements in Figure 3, the average surface roughness (R_a_) and the root-mean-square surface roughness (R_q_) were almost the same, approximately 2 nm. This shows that the UV/ozone usage did not change the surface roughness significantly.

**Figure 4.**
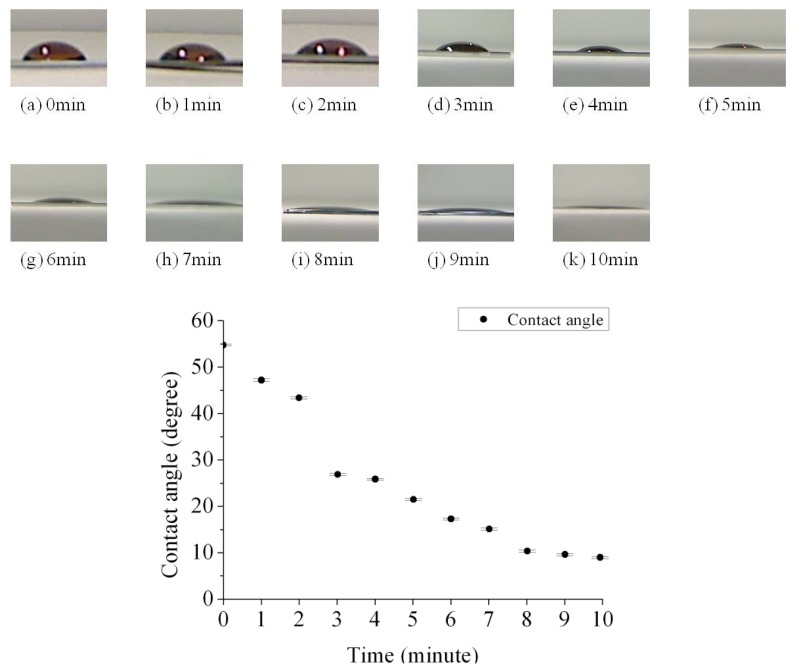
The contact angle between the protein solution and the SU-8 photoresist surface after surface modification. The contact angle decreases with increased treatment time. The surface is more hydrophilic.

We can determine how the hydrophobicity was affected by the UV/ozone process time through measuring the contact angle. The contact angles between the protein solution and the SU-8 photoresist under different UV/ozone treatment time are shown in Figure 4. We observed that the SU-8 photoresist surface became more hydrophilic after an increased UV/ozone process time. The plot summarizes the experimental result of contact angles between a protein droplet (∼10 μL) and the SU-8 photoresist layer from zero to ten minutes UV/ozone treatment. The measurement setup was based on the Sessile Drop Method [20]. The contact angle decreased monolithically with increased UV/ozone treatment time. It can be seen that after the UV/ozone process the contact angle was decreased from 55 degrees to around 10 degrees.

**Figure 5.**
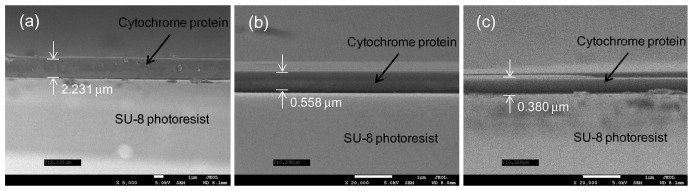
SEM pictures of the protein layer on top of the SU-8 photoresist mesa after (**a**) 3 min, (**b**) 6 min, and (**c**) 10 min UV/ozone usage time.

We selected three different UV/ozone treatment times (3, 6, and 10 min) to further investigate the effect of the UV/ozone on the performance of the cytochrome c protein onto the SU-8 photoresist. We dropped the protein solution onto the chips, which remained static for 60 s, and then rotated them at 600 rpm for 10 s. To ensure the cytochrome protein was successfully deposited on to the SU-8 photoresist mesa, we took scanning electron microscopy (SEM) pictures of the fabricated chips. The results are shown in Figure 5.

There is a clean boundary line between the cytochrome protein c and the SU-8 photoresist layer. We noticed that an increased UV/ozone treatment time on SU-8 photoresist surface leads to a thinner protein layer. The protein thickness was 2.231 μm, 0.558 μm, and 0.38 μm for each case (3, 6, and 10 min of UV/ozone treatment). This is understandable since the protein solution tends to spread more on top of the more hydrophilic surfaces. Since we dropped a fixed amount of protein solution each time, it is expected that the thinner protein layer would form for the 10 min UV treated SU-8 photoresist mesa, similar to the pictures given in Figure 4. We believe the peptides of the protein have interactions between C=O and the phenol group. These interactions provide attractive force between the cytochrome c protein and the hydrophilic SU-8 photoresist base layer.

**Figure 6.**
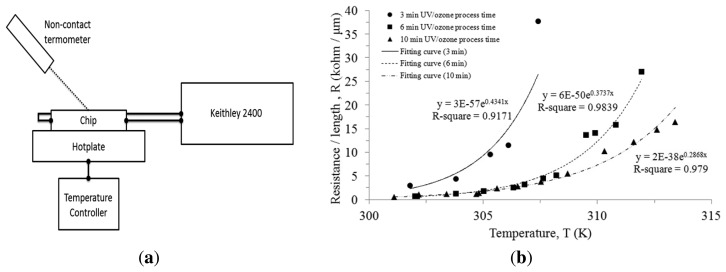
(**a**) TCR measurement setup, and (**b**) the cytochrome c thin film resistance versus temperature after 3, 6, and 9 min of UV/ozone process time on the SU-8 photoresist.

To measure the TCR performance, a chip was set on the top of a hotplate which was connected to a temperature controlling unit, as illustrated in Figure 6a. The chip surface temperature was measured with a non-contact thermometer since we were more interested in the protein surface temperature than the hotplate temperature. The two probes were connected to electrodes on the chip. The resistance of the cytochrome c thin film was not measured by direct measurement in the Keithley 2400 due to the self-heating phenomenon. To avoid continuous constant current heating the thin film, pulse voltages of 100 μs were applied at different bias to measure the current flowing through the cytochrome c thin film. A pulse voltage was given with a stop period of 100 ms, a period three orders of magnitude longer than the pulse voltage to avoid self-heating. We measured the I-V curve at various temperatures. I-V curves of the cytochrome c thin film were linear, allowing us to use the relationship to calculate resistance for each temperature.

The cytochrome c protein thin film presents an exponential increasing resistance as the temperature rises. In comparison to the chip under different UV/ozone process time, they all exhibit a similar trend. We fitted the curves and used the expression:

(1) $$\text{TCR}=\frac{1}{R}\frac{dR}{dT}$$

to calculate the TCR of the cytochrome c, where *R* is resistance and *T* is temperature. The cytochrome c thin films resistance varied with temperature and is shown in Figure 6b. There are curve fitting equations inside Figure 6b. We calculated the TCR by applying the fitting equations into Equation (1). The calculated TCR is 43%/K, 37%/K and 28%/K in each case (3, 6, and 10 min of UV/ozone treatment). It was reported that any conductive protein that has a large dipole moment when folded in its native conformation would undergo a substantial change in conductivity when the native conformation is significantly disrupted by a rise in temperature [12]. The results show a higher TCR with reduced UV/ozone treatment time since adhesion of the cytochrome protein on the SU-8 photoresist was comparably weak to the one which underwent a longer process time. The endurable temperature of the chip under 3 min of UV/ozone process is limited to approximately 307 K. This is increased to 312 K and 315 K for 6 min and 10 min UV/ozone treatment time, respectively. We found that the resistance became unstable after the temperature exceeds these values. If we continue raising the temperature, the sensors became open circuit. In other words, the longer process time could make the thin film endure higher temperatures. We believed that longer UV/ozone usage on the SU-8 photoresist surface created more C=O and the phenol group (benzene-OH) to enhance the dipole-dipole interaction. This helps the cytochrome c protein withstand higher temperatures without breaking to form an open circuit. However, the longer UV/ozone treatment also reduced the TCR value due to stronger confinement. The most stable process of 10 min UV/ozone treatment shows a TCR of approximately 28%/K, which is still much higher than conventional VO_x_ and a-Si. These experimental results demonstrated that the cytochrome c thin film could be deposited using the spin coating method on the top of the hydrophilic SU-8 photoresist surface.

## 4. Noise Performance and Stability of the Cytochrome C Protein

### 4.1. Noise Performance

**Figure 7.**
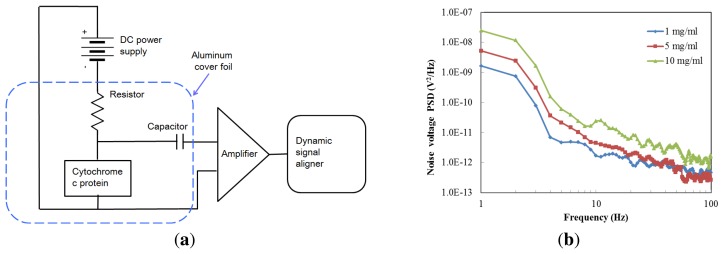
(**a**) 1/f noise measurement setup, and (**b**) frequency dependence of noise voltage at different protein concentrations.

In Figure 7a a schematic of the noise measurement setup is shown. The chip is connected in series with a low-noise DC voltage source (ABM 9603D) and a load resistance (R_L_) of 2 MΩ. A low noise current preamplifier (model SR560, Stanford Research Instruments, Sunnyvale, CA, USA) enlarged the output noise signal through a coupling capacitor (2 pF), and the resulting signal was measured with an Agilent 35670A dynamic signal analyzer (Agilent Technologies, Santa Clara, CA, USA). The noise power spectral density (PSD) was measured over the frequency range from 1 Hz to 100 Hz. The devices were placed within a low-frequency aluminum foil box to reduce the influence of extraneous noise signals. The test is under a constant bias voltage, and the voltage was set to one volt to minimize the heating effect.

The noise characteristics of cytochrome c were represented by averaging 20 runs; the results are shown in Figure 7b. We prepared three different protein concentrations in order to study the noise performance. The noise values of the cytochrome c thin film were 2.33 × 10^−13^ V^2^/Hz, 2.02 × 10^−12^ V^2^/Hz, and 2.56 × 10^−12^ V^2^/Hz, at 60 Hz with a protein concentration of 1, 5 and 10 mg/mL, respectively. These values are lower than those reported for α-Si (1.1 × 10^−6^ V^2^/Hz) [21]. The results show that a lower concentration of the cytochrome protein was less noisy.

### 4.2. Stability of Cytochrome C

**Figure 8.**
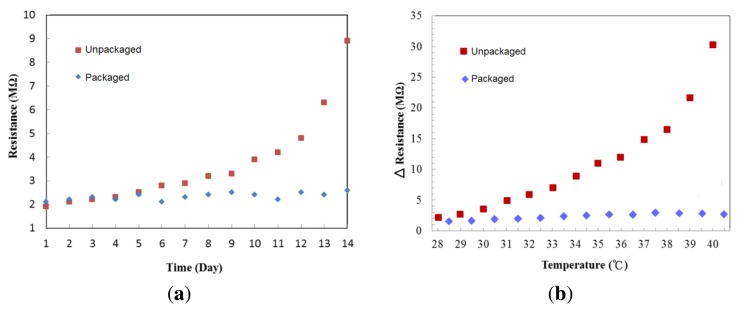
(**a**) Resistance changes in the low vacuum and ambient air environments under room temperature, (**b**) resistance difference before and after thermal cycles.

To check stability of the cytochrome protein after deposition, we purposely removed the silicon cover on top of the protein to expose it to air. Figure 8a shows the resistance changes of the cytochrome c thin films stored for two weeks under room temperature, one packaged in a low vacuum (∼10^−2^ Torr) and the other exposed to the ambient environment. The resistance changed little in the packaged device over time at room temperature. On the fourth day, the resistance of the film that was exposed to the ambient environment began to increase. We suspect this may be due to the effects of water evaporation and oxygen triggered chemical reactions on the cytochrome c protein. It is not surprising to observe this since protein denaturation can be prevented by proper packaging. To test the temperature effect on cytochrome c, we repeatedly raised the temperature in 1 °C increments, and then cooled to room temperature, 25 °C. The resistance difference before and after the thermal cycles are shown in Figure 8b. The resistance of the unpackaged device increased dramatically once the temperature was raised. In comparison, the packaged device has better stability when the temperature was less than 41 °C.

**Figure 9.**
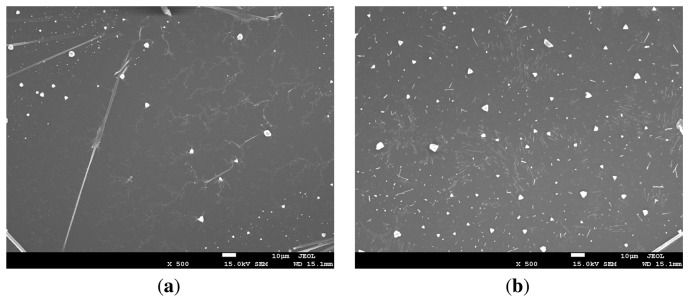
The SEM images of (**a**) fresh and (**b**) degraded cytochrome c thin film.

In Figure 9 cytochrome c thin film surfaces are shown. The film in Figure 9a was fresh and the resistance could be measured. The film in Figure 9b was heated at 45 °C for one hour in an ambient environment which made the resistance immeasurable and irreversible. It can be seen from the SEM images that the surface of a fresh protein thin layer was relatively smooth with few cracks. Under high temperature and air, the protein surface became cracked and the resistance of the thin film increased.

**Figure 10.**
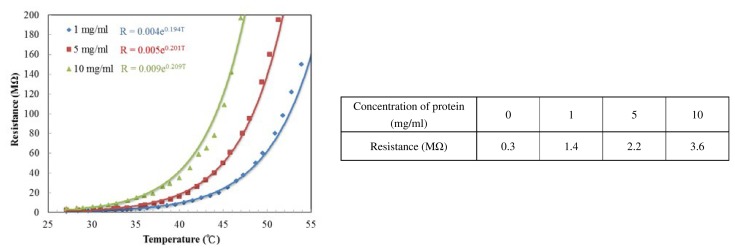
Resistance *vs.* temperature at different protein concentrations and the cytochrome c thin film resistance at different concentrations. Zero concentration of protein means that only the buffer solution was applied on the SU-8 photoresist substrate.

In order to make the IR sensing thin film, the cytochrome c must be dissolved into liquid and then spin-coated. However, when the cytochrome c is dissolved in deionized (DI) water, the resistance is too large to measure. We require a phosphate buffer solution in addition to DI water. The phosphate buffer solutions are commonly used in biological research. The resistances of different ratios of cytochrome c to phosphate buffer solution are shown in Figure 10. Omission of the cytochrome c protein powder results in the resistivity of the thin film (mainly potassium phosphate buffer solution) reducing to 0.3 MΩ. As more protein is added the resistance increased. Therefore, the phosphate buffer solution improves conductivity of the cytochrome c thin film. Figure 10 shows the experimental results, with TCR values corresponding to 19.4, 20.1 and 20.9%/K for protein concentrations of 1, 5 and 10 mg/mL, respectively. The higher the ratio of cytochrome c, the more sensitive the resistance is to temperature change. We conclude that the potassium phosphate electrolyte was responsible for conductivity for the film, while the cytochrome c protein accounts for the resistance change over temperature. It was also possible that the cracks in Figure 9b are a result of crystallization of the phosphate due to water evaporation.

Sensing materials used in bolometers should have good IR absorption, high TCR, low 1/f noise and easy deposition. We summarize different sensing materials in Table 1. The signal strength is related to the IR absorption and TCR. Cytochrome c has strong signal reception from target objects compared with other materials used in bolometers. Another advantage of the cytochrome c thin film is that it can be deposited using spin coating in a room environment, which minimizes the production costs. However, all of these advantages come at a price; the cytochrome c protein is expected to be less stable than non-organic materials. Therefore, appropriate packaging is necessary [22,23].

## 5. Conclusions

In this work, we have demonstrated how cytochrome c can be used as both an absorber and thermistor. The simulation results suggested that the high infrared absorption and the diversity of vibration frequencies of cytochrome c are due to the molecular structure. As an absorption layer, we proved experimentally that the cytochrome c protein has good IR absorption in the region 8–14 μm and is suitable for long-wavelength infrared thermal imaging. We experimentally demonstrated that the cytochrome c protein could be spin coated on a hydrophilic SU-8 photoresist surface. This simple fabrication process can provide a low cost infrared sensor in comparison to traditional methods. The TCR of the cytochrome c thin film on the SU-8 photoresist is 28%/K and is much higher than traditional materials, such as VO_x_ and a-Si. It shows great potential for infrared sensing applications. More importantly, the 1/f noise values of the cytochrome c protein can be as low as 2.33 × 10^−13^ V^2^/Hz at 60 Hz. The packaged cytochrome c protein thin film showed less degradation. The pressure inside the package has to be as low as 10^−2^ Torr for a reliable protein bolometer. We believe this work provides a new direction for developing protein-based bolometers and will allow others in this field to evolve thermography for more applications.

## Figures and Tables

**Table 1.** Comparison of different IR sensing materials.

**Material**	**Titanium**	**Amorphous Silicon**	**Vanadium Oxide**	**Yttrium Barium Copper Oxide**	**PEDOT:PSS**	**This Work**
**Type**	Metal	Semiconductor	Semiconductor	Semiconductor	Organic	Organic
**TCR (**%/**K)**	0.35 [7]	−3 [6]	−2 ∼ −3 [24]	−2.8 ∼ −4 [8]	−4 [9]	∼20
**1/f (V^2^/Hz)**	3 × 10^−15^ [25] at 1 Hz	1.1 × 10^−6^ [21] at 10 Hz	10^−12^ [26] at 0.1 Hz	2.5 × 10^−11^ [27] at 1 Hz	N/A	2.33 × 10^−13^ at 60 Hz
**IR absorption**	No	Yes	Yes	Yes	Yes	Yes
**Deposition method**	Evaporation, sputtering	Evaporation, sputtering	Evaporation, sputtering	RF-Sputtering	Spin coating	Spin coating
